# The learning curve and experience of a novel multi-modal image fusion targeted transperineal prostate biopsy technique using electromagnetic needle tracking under local anesthesia

**DOI:** 10.3389/fonc.2024.1361093

**Published:** 2024-03-11

**Authors:** Yongjun Yang, Xianya He, Yiming Zeng, Qiang Lu, Yuanwei Li

**Affiliations:** Department of Urology, Hunan Provincial People’s Hospital, The First Affiliated Hospital of Hunan Normal University, Changsha, Hunan, China

**Keywords:** prostate cancer, electromagnetic needle tracking, local anesthesia, targeted transperineal biopsy, learning curve background

## Abstract

**Background:**

Prostate cancer is the most common malignant tumor of male genitourinary system, and the gold standard for its diagnosis is prostate biopsy. Focusing on the methods and skills of prostate biopsy, we explored the learning curve and experience of a novel magnetic resonance imaging and transrectal ultrasound (mpMRI-TRUS) image fusion transperineal biopsy (TPB) technique using electromagnetic needle tracking under local anesthesia.

**Methods:**

The clinical and pathological data of 92 patients who underwent targeted TPB from January 2023 to July 2023 in our center were prospectively collected. The cumulative sum (CUSUM) analysis method and the best fitting curve were used to analyze the learning curve of this novel technique, and the clinical characteristics, perioperative data and tumor positive rate of prostate biopsy of patients at different stages of the learning curve were compared.

**Results:**

With the increase of the number of surgical cases, the overall operative time showed a downward trend. The best fitting curve of CUSUM reached its peak at the twelfth case, which is the minimum cumulative number of surgical cases needed to cross the learning curve of the operation. Taking this as the boundary, the learning curve is divided into two stages: learning improvement stage (group A, 12 cases) and proficiency stage (group B, 80 cases). The surgical time and visual analog scale score during prostate biopsy in group A were significantly higher than those in group B. The visual numerical scale score during prostate biopsy in group A was significantly lower than that in group B. There was no statistically significant difference between group A and group B in the detection rate of csPCa and the incidence of perioperative complications.

**Conclusion:**

The novel targeted TPB technique is divided into learning improvement stage and proficiency stage, and 12 cases may be the least cumulative number.

## Background

Prostate cancer (PCa) is the most common malignant tumor of male genitourinary system (1, 2). According to the 2020 GLOBOCAN statistics of the World Health Organization, there are approximately 1.4 million new cases and 375,000 deaths worldwide. PCa is the second most common tumor among male patients, second only to lung tumor, and ranks fifth among the causes of tumor death (3). At present, although prostate specific antigen (PSA), PSA density (PSAD), multi-parameter magnetic resonance imaging (mpMRI), prostate specific membrane antigen positron emission tomography/computed tomography (PSMA PET/CT) and other detection methods have played an important role in the diagnosis of PCa, but the gold standard for the diagnosis of PCa is still prostate biopsy (4–6). Compared to transrectal biopsy (TRB), transperineal biopsy (TPB) has a lower risk of infection and a higher detection rate for tumors in the tip and transitional zone of the prostate (7). The guidelines recommend that prostate biopsy should preferably be done through the transperineal route (8–10). Targeted prostate biopsy usually refers to the fusion of mpMRI image and transrectal ultrasound (TRUS) image to guide biopsy. There are three modes of mpMRI-TRUS image fusion: cognitive fusion, in-bore fusion and artificial intelligence (AI) software fusion (11). With the development of AI software image fusion technology, the detection rate of clinically significant prostate cancer (csPCa) has been significantly improved by multi-modal image fusion targeted puncture (12).

The magazine of European Urology recently reported a new image fusion targeted TPB technique using electromagnetic needle tracking under local anesthesia (LA), which can achieve a high tumor detection rate while ensuring patient comfort and low incidence of perioperative complications (13). The urology team of our hospital made further exploration on targeted biopsy and conducted a novel planar prostate biopsy: mpMRI-TRUS image fusion TPB technique using electromagnetic needle tracking under LA. In this study, the clinical and pathological data of 92 patients who underwent mpMRI-TRUS image fusion targeted TPB using electromagnetic needle tracking under LA were prospectively analyzed. By discussing the learning curve and skills of this novel technique, we can guide beginners to be familiar with the steps and key points of operation, and help them complete the growth from learning improvement stage to proficiency stage, so as to promote the wide application of this novel technique in clinical practice.

## Patients and methods

### Patients

The clinical data of 92 patients who underwent mpMRI-TRUS image fusion targeted TPB using electromagnetic needle tracking under LA from January 2023 to July 2023 in urology department of our hospital were analyzed prospectively. Inclusion criteria: ①PSA>10 ng/ml; ②suspected prostate nodules were found by digital rectal examination; ③When PSA is 4-10 ng/ml, f/tPSA<0.16, and/or PSAD>0.15 ng/ml^2^. When a prostate biopsy-naïve patient has one of the above three criteria, that is, meets the indication of prostate biopsy, the patient is further arranged to undergo mpMRI examination. The mpMRI image is used for multi-modal image fusion in subsequent prostate biopsy. Exclusion criteria: ① those who are allergic to narcotic drugs; ②there are contraindications for prostate biopsy; ③patients who had undergone transurethral resection or vaporization of prostate, or had previously received surgery and local radiotherapy for pelvic organ tumors.

All patients underwent mpMRI examination in the nuclear magnetic resonance room of our hospital before biopsy, using a 3-T system with 32-channel phased-array coil and no endorectal coil. The mpMRI sequence include morphological T2-weighted images, functional diffusion-weighted images and dynamic contrast-enhanced series images of pelvic cavity. The mpMRI scan and original image were reviewed by a uroradiologist with more than 8 years of experience, and the mpMRI sequence was evaluated according to the Prostate Imaging Report and Data System (PI-RADS) v2.1 standard (14). After the patient’s preoperative examination was completed, TPB was performed by a urologist naïve to prostate biopsy. The urologist was completely naive to TPB and had no experience with TRB. However, the urologist underwent simulator training using the novel platform before TPB and was certified in the training. Biopsy samples were collected and evaluated by a specialist uropathologist with more than 15 years of experience. According to the International Society of Urological Pathology standards, the core histology is the Gleason score and grading group (GG), with csPCa defined as GG ≥ 2.

The procedure followed in this study was in line with the requirements of the World Medical Association Declaration of Helsinki revised in 2013 (15). All patients were informed of the surgical risks and potential postoperative complications before TPB, and signed informed consent forms for anesthesia and surgical notification.

### Targeted transperineal biopsy

**Figure 1:**
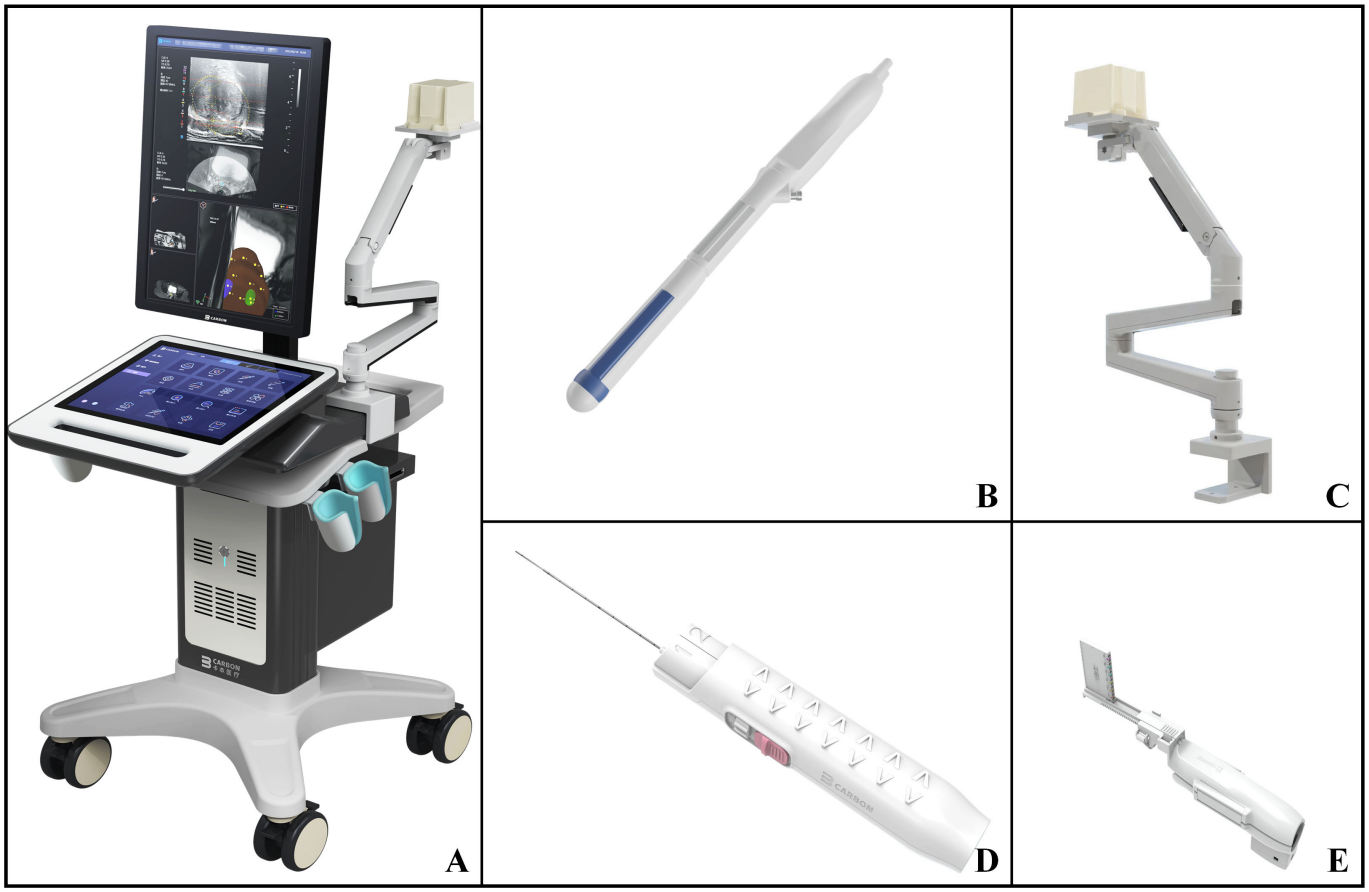
VENUS multi-modal AI image fusion ultrasound system and prostate biopsy accessories. **(A)** External view of VENUS multi-modal AI Image fusion ultrasonic system; **(B)** The intracavity biplane ultrasonic probe configured by VENUS multi-modal AI image fusion ultrasonic system; **(C)** Electromagnetic navigation and tracking system of prostate biopsy needle configured by VENUS multi-modal AI image fusion ultrasound system; **(D)** 18G disposable biopsy needle; **(E)** The disposable ultrasound probe puncture frame matched with the ultrasound probe.

The medical equipment is VENUS multi-modal AI image fusion ultrasound system produced by CARBON (Shenzhen) Medical Technology Co., Ltd. (**Figure 1A**). The ultrasound system is equipped with an intracavity biplane ultrasound probe (**Figure 1B**), which can display the cross-section and longitudinal-section ultrasound images of prostate simultaneously on the equipment screen. At the same time, the instrument is equipped with an electromagnetic navigation and tracking system for prostate biopsy needle (**Figure 1C**). The biopsy needle is produced by CARBON (Shenzhen) Medical Technology Co., Ltd. (**Figure 1D**) and the model is 18G. The electromagnetic navigation and tracking system can monitor the movement of the biopsy needle in real-time. The ultrasound probe is equipped with a disposable puncture frame (CARBON (Shenzhen) Medical Technology Co., Ltd.) (**Figure 1E**). During the process of biopsy, the puncture frame is fixed on the intracavity biplane ultrasound probe, which can stably guide the needle entry direction, and reduce the deformation of the needle tip when the needle breaks through the perineal skin.

The surgeon provides education on TPB for patients planning to undergo puncture to alleviate their nervousness. Bowel preparation was conducted by administering 40 mL of glycerin rectally 2 hours prior to biopsy, and flurbiprofen axetil injection was intravenously administered for analgesia 30 minutes before biopsy. Entering the prostate biopsy operation room, the patient takes the lithotomy position, lifts and fixes the scrotum, fully exposes the perineum. The nurse performs electrocardiogram monitoring on the patient and observes patients’ breath and SpO_2_ carefully.

The position of the electromagnetic navigation and tracking system of prostate biopsy needle is adjusted to about 10 cm above the projection of prostate body surface. The mpMRI image were imported into VENUS multi-modal AI image fusion system, and AI intelligently identified the prostate morphology and manually mapped the location of target lesions. The intracavity biplane ultrasound probe was slowly inserted into the rectum from the anal orifice. AI intelligently fused the prostate and target lesion in mpMRI image and TRUS image, and determined the puncture site of the target lesion based on the multi-modal fusion image.

**Figure 2:**
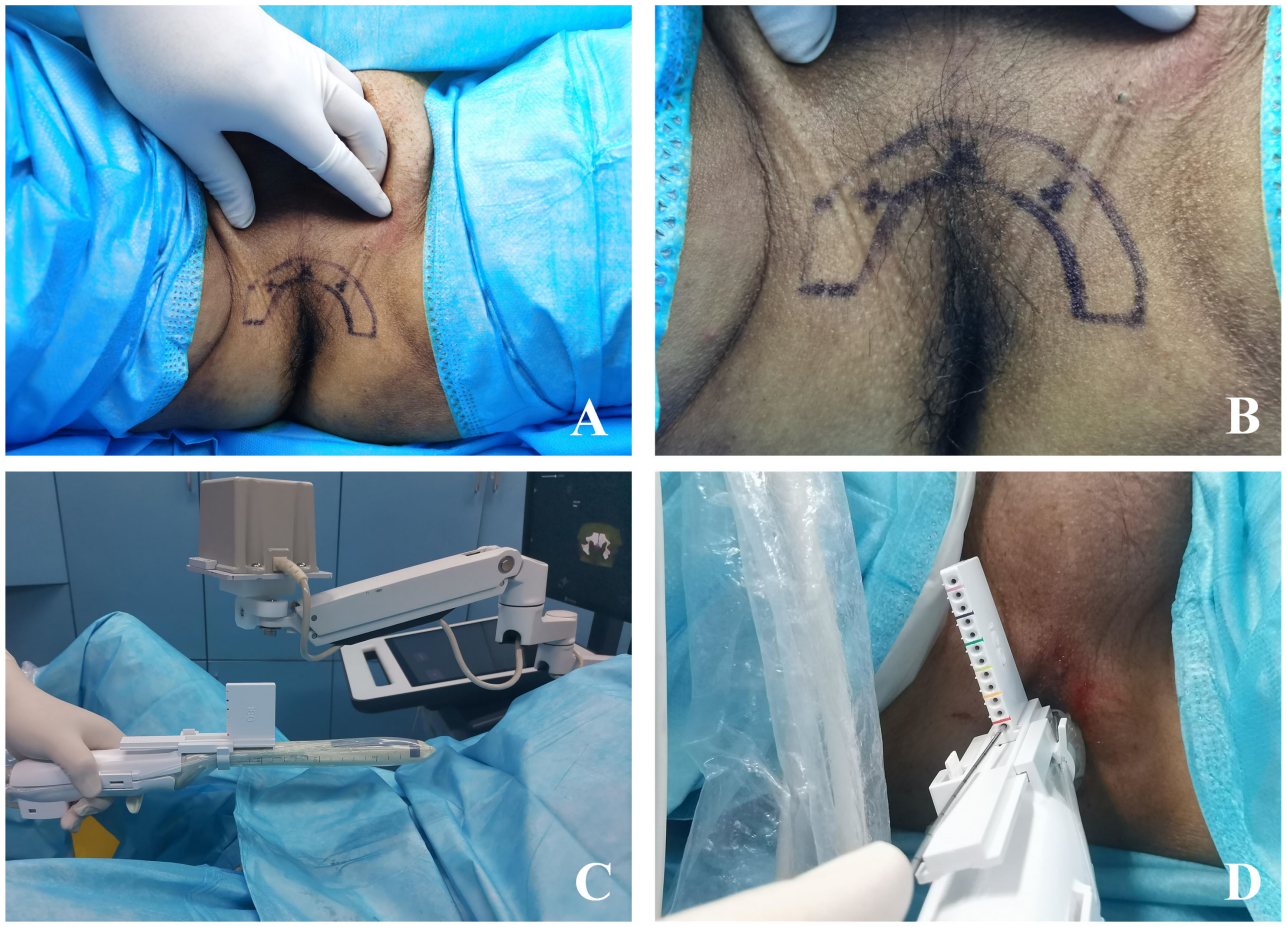
The exterior view of mpMRI-TRUS image fusion targeted TPB using electromagnetic needle tracking under LA. **(A)** One plane and three-point LA projection mark on the body surface, wherein the fan-shaped semicircle outline is the needle entry area of prostate puncture, and the three forks are the needle entry points of prostate nerve block; **(B)** Partial enlarged view of the projection mark on the body surface of one plane and three-point LA; **(C)** The ultrasound probe puncture frame is fixed on the intracavity biplane ultrasound probe, and the electromagnetic navigation tracking system is adjusted to about 10 cm above the surface projection of the prostate; **(D)** External view of targeted TPB.

**Figure 3:**
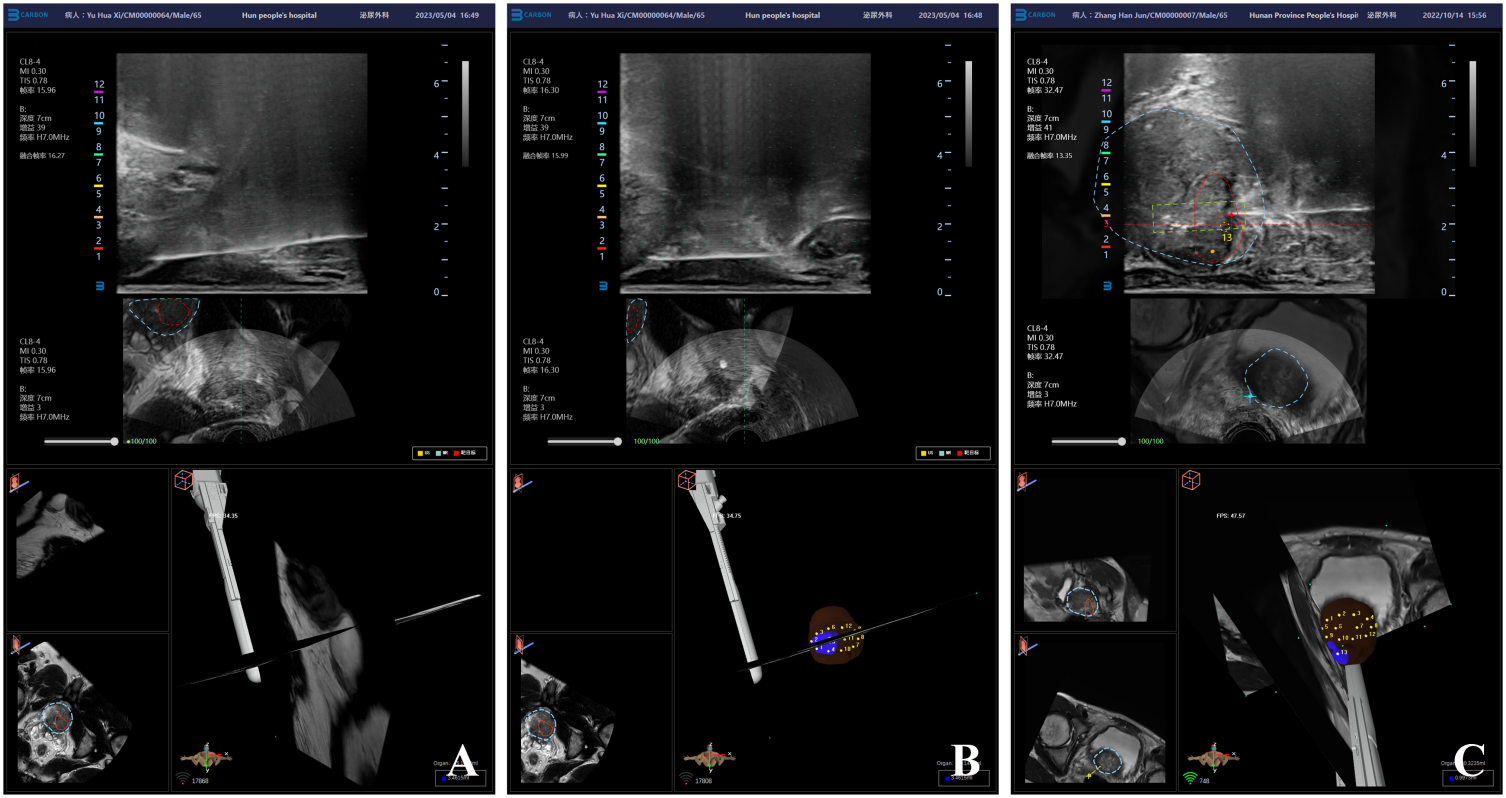
Ultrasound image of mpMRI-TRUS image fusion targeted TPB using electromagnetic needle tracking under LA. **(A)** Injection diagram of local anesthetic under the capsule at the tip of prostate; **(B)** Local anesthetics injection map under the capsule on the left and right sides of the prostate; **(C)** mpMRI-TRUS image fusion guided targeted TPB, the red area is the target lesion, and the yellow rectangular frame area is the starting point and end point of real-time monitoring of biopsy needle emission by electromagnetic navigation and tracking system.

Biplane ultrasound probe was used to locate “one planar and three-point” LA. “One planar” refers to applying compound lidocaine cream on the perineal skin for surface anesthesia, while injecting lidocaine hydrochloride subcutaneously into the perineum for superficial infiltration anesthesia to reduce the pain caused by the biopsy needle penetrating the skin. 10.0 mL of 2% lidocaine hydrochloride was diluted with 10.0 mL of physiological saline, and then 10.0 mL of diluted anesthetic was extracted for superficial infiltration anesthesia (**Figures 2A, B**). As for “three-point”, the needle is inserted into the tip, left and right side of prostate for peripheral nerve block anesthesia. Biplane ultrasound image is used to guide LA, and the process of anesthetic injection and diffusion is monitored simultaneously on both coronal and sagittal planar of prostate to ensure the analgesic effect of LA. Under the guidance of ultrasound image, the longer syringe needle (0.7×80 mm, Zhejiang Kangdelai Medical Devices Co., Ltd.) penetrates vertically into the tip of prostate. Then the diluted anesthetic drug was injected slowly, and the hypoechoic protuberance under the capsule of prostate could be seen on the ultrasound image (**Figure 3A**). Previous research indicates that the sensitivity to pain is higher in the prostate-tip compared to other areas, and thus, LA specifically targeting this region achieves lower pain scores possibly due to its higher nerve density (16). In order to avoid anesthetic drug entering the blood, the syringe shall be drawn back before each injection to ensure that there is no blood return. In the same way, the diluted anesthetic drug was injected under the capsule of the left and right sides of the prostate for peripheral nerve block anesthesia (**Figure 3B**). After successful anesthesia, the puncture frame was fixed on the biplane ultrasound probe (**Figure 2C**) and slowly pushed into the rectum (**Figure 2D**). Under the guidance of electromagnetic needle tracking system, a urologist naïve to prostate biopsy performed targeted biopsy combined with systematic biopsy (**Figure 3C**).

**Figure 4:**
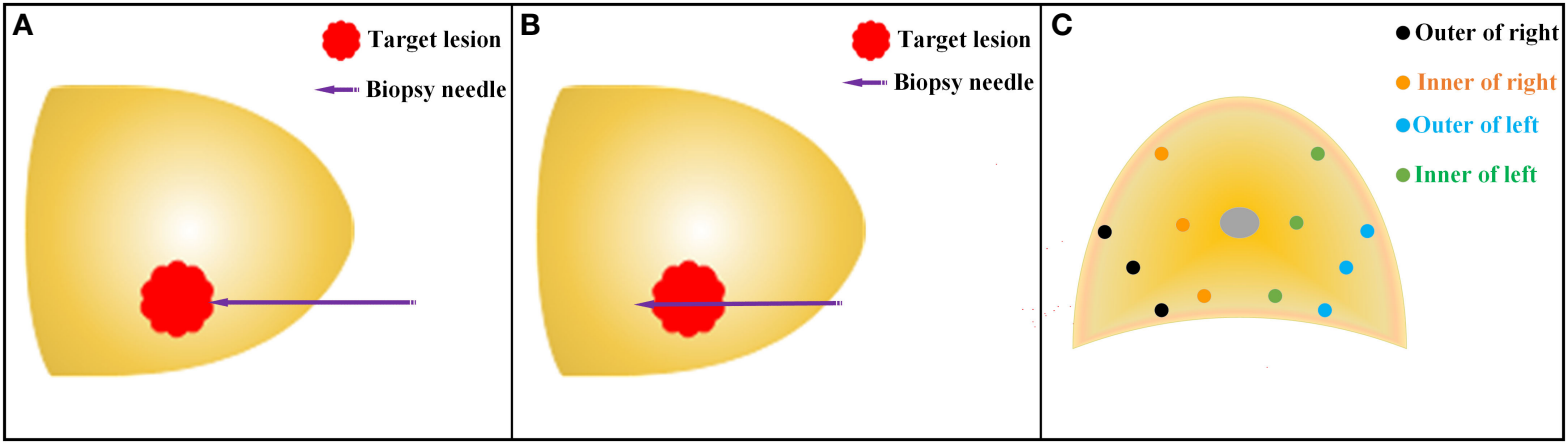
Schematic diagram of TPB. **(A)** Before the release of the biopsy needle, the needle tip reaches the outer edge of the target lesion under the guidance of the electromagnetic needle tracking system; **(B)** After the release of the biopsy needle, the needle tip penetrates through the target lesion. **(C)** Schematic diagram of systematic prostate biopsy.

A novel planar prostate biopsy technique was adopted. First, the prostate target lesions were biopsied. AI image fusion technology intelligently fuses multi-modal mpMRI-TRUS image and displays prostate target lesions in real-time. Under the guidance of electromagnetic needle tracking, the puncture frame is fixed on the biplane ultrasound probe to guide the biopsy needle into the outer edge of the target lesion (**Figure 4A**). When trigger the launch button, the needle performs “penetrating” precise puncture in the target lesion (**Figure 4B**). Subsequently, intracavity biplane ultrasound probe guided systematic biopsy was performed (**Figure 4C**). After the fusion of mpMRI and TRUS image, rotate the biplane ultrasound probe along its long axis to observe the route of the urethra and the position of the lateral margins of the left and right lobes of the prostate. Avoid lifting or pressing the ultrasound probe during the prostate biopsy, so as to reduce the contour deformation or position deviation during operation. The ultrasound probe was rotated longitudinally to the outer gland of the right prostate, and the posterior, middle, and anterior points were biopsied through the holes in the puncture frame from bottom to top. The biopsy samples were named right outer posterior, right outer middle, and right outer anterior for pathological examination. Then the ultrasound probe was rotated longitudinally to the middle position to observe the route of the urethra, and then slightly deflect the ultrasound probe to the right to prevent the needle from penetrating the urethra and causing postoperative gross hematuria. The biopsy needle passes through the needle holes in the puncture frame from bottom to top sequentially, and punctures the posterior, middle, and anterior points of the inner gland of the right prostate. The biopsy samples were named right inner posterior, right inner middle, and right inner anterior for pathological examination. A 6-needle systematic biopsy was performed on the left grand of the prostate using the same method.

### Observation indicators

The visual analog scale (VAS) was used to evaluate the degree of pain: a score of 0 was painless, with no pain sensation; 1-3 points were mild pain and do not affect daily life; 4-6 points were severe pain and affect work and life; 7-10 points were severe pain and seriously affect work and life. The visual numerical scale (VNS) was used to evaluate the satisfaction of LA effect: a score of 0 was unsatisfactory, a score of 1 was average, a score of 2 was satisfactory, a score of 3 was relatively satisfactory, and a score of 4 was very satisfactory. Preoperative education on the pain of prostate biopsy and VAS and VNS training for patients. During the surgery, the patient was scored by the same doctor using a scoring scale, and the scores of pain and satisfaction during puncture were expressed using VAS-1 and VNS-1.

Clinical data related to perioperative period were recorded. The operative time (OT) (LA time+prostate biopsy time) and perioperative complications were recorded, including hematuria, perineal hematoma, urinary tract infection, urinary retention, hemospermia, vagus nerve reaction and septic shock. Pathological results of prostate biopsy were collected after operation.

### CUSUM analysis

Graphpad Prism 9.0 statistical software was used to arrange the OT according to the order of operation date. The CUSUM_1_ value of the first case is the difference between the puncture operative time OT_1_ of the first case and the average operative time OT_mean_ of all cases, that is, CUSUM_1_=(OT_1_-OT_mean_). The CUSUM_n_ value in the second and subsequent cases is the difference between the operative time OT_n_ and the average operation time OT_mean_, plus the CUSUM_(n-1)_ value of the previous case, that is, CUSUM_(n)_=(OT_n_-OT_mean_)+CUSUM_(n-1)_, which is accumulated continuously according to this rule until the last case CUSUM approaches 0 (17).

### Learning curve fitting

The scatter plot of the learning curve was drawn with the number of surgical cases as the abscissa and the CUSUM value as the ordinate, and the CUSUM learning curve was fitted by Excel software. The goodness of fit is judged by the coefficient R². The closer R² is to 1, the higher the goodness of fit is, and the model with the highest R² is the best fitting model. Taking the vertex of CUSUM fitting curve as the boundary, the learning curve is divided into two stages: learning improvement stage (group A) and proficiency stage (group B). The abscissa value corresponding to the vertex of the fitting curve is the minimum number of surgical cases that need to be accumulated to cross the learning curve.

### Statistical analysis

The clinical and pathological data of patients were analyzed by Graphpad Prism 9.0 statistical software. Continuous variables are reported as the mean ± standard deviation after a test for normality, and the comparison between the two groups uses independent sample t-test. Results for categorical variables are summarized using the absolute frequency and percentage. A χ^2^test (Fisher’s exact test) was used to identify statistically significant differences between two groups, with significance set at P<0.05.

## Results

### CUSUM learning curve analysis

**Figure 5:**
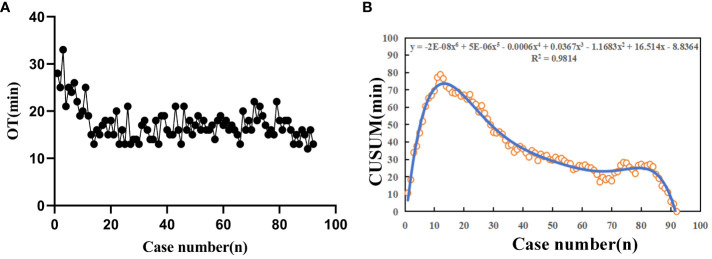
The learning curve diagram of multi-modal image fusion targeted TPB using electromagnetic needle tracking under LA. **(A)** OT shows an overall downward trend as the number of surgical cases increases; **(B)** Scatter diagram of CUSUM learning curve for TPB. The blue thin line represents the best fit model of sixth power curve, and the corresponding abscissa of the fitting curve vertex is 12 cases.

All patients successfully completed the mpMRI-TRUS image fusion targeted TPB using electromagnetic needle tracking under LA. The OT was (17.35 ± 3.67) min. The longest OT was 33 min (the prostate volume of this patient was 77.46 cm^3^, the body mass index (BMI) value was 33.57%, and LA took a long time). The shortest OT was 12 min. As can be seen from the trend chart of OT, with the increase of the number of surgical cases, the overall OT shows a downward trend (**Figure 5A**). By fitting the scatter plot with the number of surgical cases as abscissa and the OT CUSUM value as ordinate, it is judged that the best fitting model is a sixth power curve, the fitting goodness coefficient R² is 0.9814, and the fitting equation is CUSUM(min) =−2E-08X^6^+5E−06X^5^-0.0006X^4 +^ 0.0367X³−1.1683X²+16.514X−8.8364 (X represents the number of surgical cases). The fitting curve crosses the vertex when the number of surgical cases accumulates to the 12th case. Taking this as the boundary, the learning curve is divided into two stages: A stage is the learning improvement stage, B stage is the proficiency stage, and 12 cases are the minimum number of surgical cases that need to be accumulated to cross the learning curve (**Figure 5B**).

### Comparison of general data of patients in two stages of learning curve

The general information of patients in the two stages is shown in **Table 1**. There was no statistically significant difference between the learning improvement stage group A and the proficiency stage group B in terms of patient age (68.83 ± 7.70 and 68.25 ± 7.98, *P*=0.8132), prostate volume (61.94 ± 30.04 and 51.37 ± 25.92, P=0.2003), blood tPSA (25.33 ± 19.10 and 24.89 ± 22.59, *P*=0.9491), and PI-RADS score (4.08 ± 1.17 and 3.78 ± 1.18, *P*=0.4000).

**Table 1:** Comparison of preoperative general data of patients in two stages of learning curve.

	A(n=12)	B(n=80)	*P*
Age (years)	68.83 ± 7.70	68.25 ± 7.98	0.8132
Prostate volume (cm^3^)	61.94 ± 30.04	51.37 ± 25.92	0.2003
Total PSA (ng/ml)	25.33 ± 19.10	24.89 ± 22.59	0.9491
PI-RADS score	4.08 ± 1.17	3.78 ± 1.18	0.4000

### Analysis of perioperative results of patients with two stages of learning curve

The perioperative results of patients in two stages are shown in **Table 2**. The OT value (23.92 ± 4.01) and VAS-1 score (2.25 ± 0.62) in the learning improvement stage group A were significantly higher than those in the proficiency stage group B (16.36 ± 2.37 and 1.54 ± 0.62, *P*<0.001). The VNS-1 score in group A was significantly lower than that in group B (2.25 ± 0.45 and 2.75 ± 0.44, *P*<0.001). There was no statistically significant difference between group A and group B in the detection rate of csPCa [58.33% (7/12) and 65.00% (52/80), *P*=0.7499] and the incidence rate of complications [41.67% (5/12) and 18.75% (52/80), *P*=0.1254].

**Table 2:** Comparison of perioperative data of patients in two stages of learning curve.

	A (n=12)	B (n=80)	*P*
OT (min)	23.92 ± 4.01	16.36 ± 2.37	<0.001
VAS-1	2.25 ± 0.62	1.54 ± 0.62	<0.001
VNS-1	2.25 ± 0.45	2.75 ± 0.44	<0.001
Tumor detection rate (n, %)	7 (58.33%)	52 (65.00%)	0.7499
Perioperative complications (n, %)	5 (41.67%)	15 (18.75%)	0.1254
Hematuria	4 (33.33%)	13 (16.25%)	
Urinary retention	1 (8.34%)	2 (2.5%)	
Perineal hematoma	0 (0.00%)	0 (0.00%)	
Urinary tract infection	0 (0.00%)	0 (0.00%)	
Hemospermia	0 (0.00%)	0 (0.00%)	
Vagus nerve reaction	0 (0.00%)	0 (0.00%)	
Septic shock	0 (0.00%)	0 (0.00%)	

## Discussion

With the aggravation of the aging of the population in China, the incidence rate of PCa has jumped to the first place in genitourinary system tumors, which has become an important cause of serious threat to the health of Chinese people (2). Prostate biopsy is the gold standard for diagnosing PCa. According to the difference in the position of the prostate puncture needle entering the human body, there are currently two main methods: TRB and TPB. Compared to TRB, transperineal prostate biopsy has fewer perioperative complications and a higher detection rate for tumors in the tip and transitional zone of the prostate (18, 19). Relevant guidelines recommend that transperineal prostate puncture should be the first choice for biopsy of patients with suspected PCa (8–10).

The anesthesia methods used in prostate biopsy include general anesthesia, spinal anesthesia and LA of prostate. Before general anesthesia, the patient’s anesthesia tolerance should be fully evaluated, which leads to a certain degree of extension of hospitalization time and increase hospitalization expenses (20). In order to optimize the anesthesia process, our team began to explore the use of one plane and three-point biplane ultrasound to locate LA for prostate biopsy. In order to improve the LA effect of prostate biopsy, intravenous analgesics were used before operation to relieve the pain of perineal prostate biopsy under the anesthesia of peripheral prostate nerve block to the greatest extent (21). Similar to TRB, compound lidocaine cream was pushed into the rectum before operation for rectal mucosal surface anesthesia to reduce the discomfort caused by the movement of the ultrasonic probe in the intestine. Subcutaneous infiltration anesthesia was performed in the perineal puncture area to alleviate the pain caused by the penetration of the prostate biopsy needle through the perineal skin. Compared with simply inserting needles into the left and right sides of the prostate for nerve block anesthesia (13), our study added nerve block anesthesia to the tip of the prostate, which further improved the pain and satisfaction scores of patients undergoing transperineal prostate biopsy.

In order to improve the accuracy of prostate biopsy and reduce the perioperative complications of prostate biopsy, our team applied a novel multi-modal image fusion targeted transperineal prostate biopsy technique using electromagnetic needle tracking under LA. In China, due to the relative shortage of PSMA PET/CT equipment, it takes a long time for suspected PCa patients to make an appointment for PSMA PET/CT examination, and the cost is relatively high. At present, the fee is about 1,500 dollars. However, MRI equipment is relatively popular and the cost is relatively low. At present, the cost is about 150 dollars, which is about one tenth of the cost of PSMA PET/CT examination. Meanwhile, the meta-analysis found that mpMRI is comparable to PSMA PET/CT in terms of prostate tumor localization and staging detection performance (22). The multi-modal image fusion targeted transperineal prostate biopsy based on mpMRI mainly includes mpMRI-TRUS cognitive fusion, in-bore fusion and AI software fusion. The cognitive fusion targeted biopsy process is greatly influenced by subjective factors and the surgeon’s level of film reading, which may lead to missed diagnosis (23, 24). In this study, mpMRI images and TRUS images are intelligently elastic fused with the help of the software provided by the multi-modal AI image fusion ultrasound system, which reduces the operator’s requirements for mpMRI images reading ability and improves the accuracy of targeted puncture. Similar to prostate template mpMRI-TRUS fusion targeted puncture (25), this study uses a disposable ultrasound probe puncture frame to stabilize the position and direction of biopsy needle during puncture and reduce the incidence of perioperative complications. At the same time, compared with the intracavity single-plane ultrasound probe, using the biplane ultrasound probe to guide prostate puncture has a better display effect of urethral ultrasound image during puncture, and the incidence of postoperative hematuria is significantly reduced (79.00% vs. 19.75%) (13). The guidance function using electromagnetic needle tracking can visually display the puncture direction of disposable biopsy needle in prostate and the sampling length of prostate biopsy, so as to realize “penetrating” accurate puncture of target lesions and avoid the side damage of tissues around prostate caused by misjudgment of needle-tip puncture length. Our cancer detection rate was consistent with previous published data on TPB done under LA (64.13% vs. 63.33%). Regarding perioperative complications, consistent with previous studies, no patients experienced infectious-related complications, and the prevalence of postoperative acute urinary retention was comparable (26). There was no significant difference in the incidence of perioperative complications and the positive rate of tumor puncture between the learning improvement stage (group A) and the mastering stage (group B), which indicated that the multi-modal image AI fusion targeted transperineal prostate biopsy technique using electromagnetic needle tracking under LA had comparable accuracy and safety in different stages of learning curve.

Scholars have conducted relevant research on prostate puncture skills and learning curve. Transrectal prostate biopsy under epidural anesthesia, the operator may need to accumulate at least 42 cases to master the real-time fusion of MRI and TRUS images targeted prostate biopsy technique (27). When the cumulative number of cases reached 98, the accuracy of targeted prostate biopsy was significantly improved (28). With the help of prostate positioning template, it is easy to lay out the prostate needle site during the operation, and the learning curve of prostate puncture for urologists without prostate biopsy experience only needs about 18 cases (29). In the process of targeted prostate biopsy, our team fixed the disposable ultrasound probe puncture frame on the biplane ultrasound probe, and intelligently fused and calibrated the mpMRI image and TRUS image through the multi-modal AI image fusion ultrasound system, so as to quickly guide the direction of prostate puncture and monitor the moving position of the biopsy needle tip in real-time. The results of our study show that the targeted TPB technique using electromagnetic needle tracking under LA is convenient and simple, and it is easy for beginners to quickly leap from learning improvement stage to proficiency stage.

Our findings must be interpreted in the context of the study design. First, being a retrospective study, there may have been selection bias. However, the completeness of electronic medical record data including surgical records, perioperative complications and histopathological reports ensured credible results. Second, generalizability needs to be considered due to changes in the characteristics of practitioners performing prostate biopsy. An increasing number of non-urologic practitioners, including physicians, radiologists, and interventional radiologists, are performing this technique. Third, the success of targeted TPB depends on the combined performance and expertise of radiologists, urologists, and pathologists, as well as the platform used. There is a learning curve in mpMRI reading by radiologists and in the Gleason score of prostate specimens by pathologists. Finally, a better arbiter of biopsy quality may be core length of the biopsy and the presence of stroma/skeletal muscle. In the future, prospective clinical trials will be conducted to better verify the relevant conclusions of this study. Despite these limitations, this study provides added insight into the learning curve and experience of a novel multi-modal image fusion targeted TPB technique using electromagnetic needle tracking under LA.

## Conclusion

This study explored the learning curve and clinical experience of a novel multi-modal image fusion targeted TPB technique using electromagnetic needle tracking under LA for the first time. AI image elastic fusion targeted biopsy weakens the dependence of operators on mpMRI reading ability. Patients under LA have good tolerance to biopsy, and the pathological results suggest a relatively high positive diagnosis rate of csPCa. It is worth popularizing and applying in most hospitals. From the fitting curve of CUSUM analysis method, it can be found that after 12 cases of prostate biopsy with multi-modal mpMRI/TRUS image fusion, the operator crossed the learning curve from the learning improvement stage to the proficiency stage, and the operation time, pain score and satisfaction score during prostate biopsy tended to be stable.

## Data availability statement

The original contributions presented in the study are included in the article/supplementary material. Further inquiries can be directed to the corresponding authors.

## Ethics statement

The studies involving humans were approved by Ethics Committee of Hunan Provincial People’s Hospital. The studies were conducted in accordance with the local legislation and institutional requirements. The human samples used in this study were acquired from primarily isolated specimens as part of a previous study for which ethical approval was obtained. Written informed consent for participation was not required from the participants or the participants’ legal guardians/next of kin in accordance with the national legislation and institutional requirements.

## Author contributions

YY: Conceptualization, Data curation, Formal Analysis, Investigation, Methodology, Project administration, Software, Supervision, Validation, Writing – original draft, Writing – review & editing. XH: Conceptualization, Data curation, Investigation, Methodology, Software, Writing – original draft. YZ: Data curation, Formal Analysis, Methodology, Project administration, Supervision, Writing – original draft. QL: Formal Analysis, Methodology, Project administration, Supervision, Validation, Writing – original draft. YL: Conceptualization, Funding acquisition, Investigation, Resources, Software, Visualization, Writing – original draft, Writing – review & editing.
